# Hanging drop cathode-atmospheric pressure glow discharge as a new method of sample introduction for inductively coupled plasma-optical emission spectrometry

**DOI:** 10.1007/s00216-020-02685-7

**Published:** 2020-05-11

**Authors:** Krzysztof Swiderski, Maja Welna, Krzysztof Greda, Pawel Pohl, Piotr Jamroz

**Affiliations:** Faculty of Chemistry, Department of Analytical Chemistry and Chemical Metallurgy, Wroclaw University of Science and Technology, Wybrzeze Stanislawa Wyspianskiego 27, 50-370 Wroclaw, Poland

**Keywords:** Hanging drop cathode, Atmospheric pressure glow discharge, Inductively coupled plasma, Optical emission spectrometry, Sample introduction

## Abstract

This work reports the use of hanging drop cathode-atmospheric pressure glow discharge (HDC-APGD) as a new method of sample introduction for inductively coupled plasma-optical emission spectrometry (ICP-OES). The developed arrangement was characterized by a low sample uptake (0.56 mL min^−1^) and the fact that the entire sample solution volume was consumed by the discharge. This resulted in a very high transport efficiency of analytes from the sample solution into the ICP torch (usually > 80%). Under the optimal operating conditions of HDC-APGD, intensities of emission lines of studied elements were, on average, 2 times higher as compared to those obtained with conventional pneumatic nebulization (PN). Moreover, in the case of I and Y, the observed signal enhancements were even higher, i.e., 6.2 and 6.1 times, respectively. It was also shown that in the case of B and some elements that are known to form different volatile species (Ag, Bi, Cd, Hg, Os, Pb, and Se), the presence of low molecular weight organic compounds in the sample solution, i.e., CH_3_OH, C_2_H_5_OH, HCOOH, CH_3_COOH, or HCHO, resulted in the additional enhancement of their signals. It was especially evident in the case of Hg for which a 8.6-fold signal enhancement in the presence of HCOOH was noticed. The system presented herein was distinguished from other competitive APGD-type discharges because it could be successfully used for the determination of a vast group of elements, including alkali metals, alkaline earth metals, transition metals, and non-metals.

**Electronic supplementary material:**

The online version of this article (10.1007/s00216-020-02685-7) contains supplementary material, which is available to authorized users.

## Introduction

Inductively coupled plasma-optical emission spectrometry (ICP-OES) is one of the most widely used analytical methods for multi-element analysis of liquid samples due to the high precision of measurements, broad linearity ranges of calibration curves, and low limits of detection (LODs) for the majority of elements. Unfortunately, ICP-OES is not free from weaknesses, which mainly include the way the sample is introduced into the ICP spectrometer. The best-known and conventionally applied sample introduction system, based on pneumatic nebulization (PN), provides a quite low transport efficiency of analytes into a plasma source (typically up to 5%) [1]. Therefore, it is not surprising that alternative sample introduction techniques are being developed, e.g., ultrasonic nebulization (USN) [2–5], hydride generation (HG) [5–8], and photochemical vapor generation (PVG) [8–10]. Relatively new approaches, based on electric discharge phenomena, are also increasingly appreciated, including dielectric barrier discharge (DBD) [11], electrolyte-as-cathode glow discharge (ELCAD) [12], and other ELCAD-derived microplasmas, e.g., solution cathode glow discharge (SCGD) [13–15].

For the first time, an ICP spectrometer was combined with an ELCAD system by Cserfalvi and Mezei in 2005 [12]. In this approach, the electric discharge was generated in contact with an analyzed bulky sample solution, and the resulted aerosol flux, containing water vapor and sputtered elements atoms, was subsequently transferred into the ICP torch in order to investigate the sputtering mechanism of the liquid cathode. As compared to conventional PN-ICP-OES, the response of most of the studied elements in the ELCAD-ICP-OES coupled system was apparently lower; however, in the case of Hg, the intensity of the Hg I at 253.7 nm emission line was 17 times higher, likely due to Hg cold vapor generation. In 2008, Zhu et al. combined ICP-OES with SCGD for the determination of Hg as well [13]. Under the influence of the discharge, the Hg cold vapor was generated from the sample solution and swept by an Ar flow into the ICP torch, resulting in a 16-fold improvement of the analytical signal. Furthermore, they found that the intensity of the Hg emission line was even higher by a factor of 2–3 in the presence of low molecular weight organic acids and alcohols added to the sample solution. It is worth to mention that the SCGD system was also successfully used for generation of the volatile species of Os [14] and I [15] that were subsequently introduced to an ICP-OES spectrometer. The biggest shortcoming of the abovementioned systems was that high solution flow rates were typically required to sustain the discharge (from 1.2 to 10 mL min^−1^) and that only a small part of the introduced sample solution was transported into the plasma.

Quite recently, we presented a novel microplasma system based on the atmospheric pressure glow discharge (APGD) operated with a renewable hanging drop cathode (HDC) [16]. In comparison to ELCAD or SCGD, HDC-APGD required a much lower sample flow rate (~ 0.4 mL min^−1^) and provided complete evaporation of the sample solution. Moreover, the advantage of HDC-APGD system was self-ignition of the discharge [16]. Taking into account a small sample uptake rate, it can be hypothesized that HDC-APGD is more suitable for the transport of analytes than ELCAD, SCGD, or a conventional pneumatic nebulizer/spray chamber system. Hence, the aim of the present work was to preliminary evaluate the analytical response of ICP-OES combined with a new sample introduction system based on HDC-APGD. The optimization of operating parameters of HDC-APGD, i.e., carrier gas flow rate, discharge current, and pH of the sample solution, was carried out. Subsequently, the effect of the addition of low molecular weight organic compounds (LMWOCs), i.e., formic acid, acetic acid, methanol, ethanol, and formaldehyde, into the sample solution was investigated in detail. Under the optimal working conditions of HDC-APGD-ICP-OES, the background corrected intensities of emission lines of 47 elements (i.e., Ag, Al, As, B, Ba, Be, Bi, Ca, Cd, Co, Cr, Cu, Dy, Er, Eu, Fe, Ga, Ge, Hg, Ho, I, In, Ir, K, Li, Mg, Mn, Na, Nb, Ni, Os, Pb, Pd, Pr, Pt, Rb, Rh, Sb, Sc, Se, Sn, Sr, Tb, Tl, V, Y, and Zn) were determined and compared with those obtained for conventional PN-ICP-OES.

## Materials and methods

### Instrumentation

**Fig. 1:**
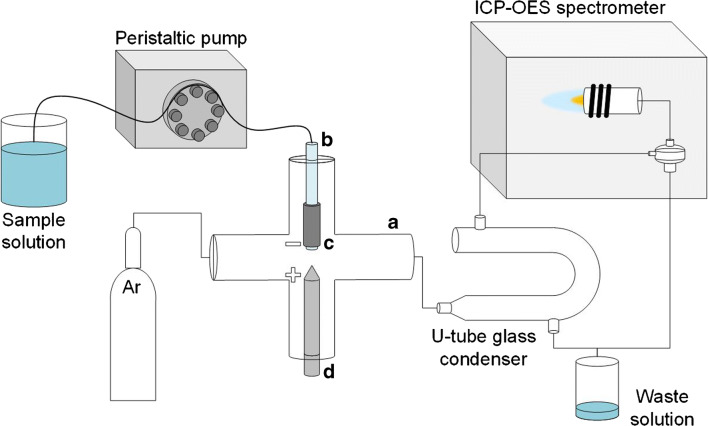
A scheme of HDC-APGD-ICP-OES system: (A) glass discharge chamber, (B) delivering quartz tube, (C) graphite tube, (D) tungsten rod

A scheme of the HDC-APGD-ICP-OES experimental setup is given in Fig. 1. The analyzed sample solution was introduced by a REGLO ICC3 ISM 4312 peristaltic micro-pump (Ismatec, USA) into a glass discharge chamber (A). The sample solution was delivered to the discharge chamber through a quartz tube (*ø*_in/out_ = 1/3 mm) (B) that was tightly wrapped by a graphite tube (*ø*_in/out_ = 3/6 mm) (C). The introduced solution spilled out of the quartz tube and formed a renewable hanging drop that was consumed by the sustained discharge. Just below the hanging drop, there was a tapered tungsten rod (*ø* = 4 mm) (D). The distance between the tip of the tungsten rod (anode) and the surface of the hanging drop (cathode) was 3.5 mm, and it was determined as the longest distance providing self-ignition and stable operation of the discharge. Using platinum wires attached to the graphite tube and the tungsten rod, a high voltage (1.0–1.5 kV) was supplied to the discharge compartment by a direct current supply (Dora, Poland). A 10 kΩ ballast resistor was connected in a series to stabilize the discharge. As the discharge was sustained, the sample solution was totally sputtered and/or evaporated, while the resulting aerosol flux, containing evaporated water molecules and sputtered analytes atoms, was swept by an Ar flow to a cyclonic chamber, and subsequently to a torch of the axially viewed ICP-OES spectrometer (model 720, Agilent, USA). Based on the weight of the waste solution collected from the cyclonic chamber, it was found that 53 ± 2% (m/m) of the sample solution was introduced into the ICP torch (carrier Ar flow rate = 0.75 L min^−1^, HDC-APGD current = 60 mA). Such a high amount of the water vapor flux affected the stability of ICP and even caused extinguishing of the plasma. To reduce the amount of water introduced into ICP, a U-tube glass condenser was placed between the discharge compartment and the cyclonic chamber. The dimension of U-tube was about 25 cm in length and 8 mm in internal diameter. The excess of the water vapor condensed inside the U-tube condenser was drained to wastes. It was found that using the U-tube condenser only 17 ± 1% (m/m) of the sample solution was transferred into ICP, which resulted in its stable operation.

The intensity of the atomic emission lines of Ag, Al, As, B, Be, Bi, Ca, Cu, Ga, Ge, I, In, K, Li, Mg, Na, Pd, Pt, Rb, Rh, Sb, Se, Sn, Tl, and Zn at 328.1, 396.2, 193.7, 249.7, 234.9, 223.1, 422.7, 327.4, 294.4, 265.1, 178.2, 325.6, 766.5, 670.8, 285.2, 589.6, 340.5, 214.4, 780.0, 343.5, 206.8, 196.0, 284.0, 351.9, and 213.9 nm, respectively, and the ionic lines of Ba, Cd, Co, Cr, Dy, Er, Eu, Fe, Hg, Ho, Ir, Mn, Nb, Ni, Os, Pb, Pr, Sc, Sr, Tb, V, and Y at 455.4, 226.5, 238.9, 267.7, 353.2, 349.9, 420.5, 238.2, 194.1, 345.6, 212.7, 257.6, 269.7, 231.6, 225.6, 220.4, 410.1, 361.4, 407.8, 350.9, 292.4, and 371.0 nm, respectively, were measured. Background corrected intensities of the emission lines of elements acquired using HDC-APGD-ICP-OES were compared to those obtained with the same ICP-OES spectrometer but operated with a sample introduction system based on a single-pass cyclonic spray chamber (Agilent, USA) and a concentric OneNeb pneumatic nebulizer (PN) (Agilent, USA). The ICP spectrometer was operated using the parameters recommended by the instrument manufacturer (see Electronic Supplementary Material (ESM) Table S1). ICP Expert II software was used for handling the spectrometer and for the data acquisition and its re-processing. All measurements were made in three replicates (*n* = 3), and average values were taken into consideration.

### Reagents and sample preparation

Deionized water was used throughout. To obtain a proper value of pH of sample solutions, an ACS grade concentrated 65–68% (m/m) HNO_3_ solution (Sigma-Aldrich, Darmstadt, Germany) was used. Solutions of LMWOCs, i.e., 100% (m/m) CH_3_OH, 96% (m/m) C_2_H_5_OH, 85% (m/m) HCOOH, 99% (m/m) CH_3_COOH, and 40% (m/m) HCHO (Avantor Performance Materials, Gliwice, Poland) were used to modify the composition of working standard solutions. Single-element standard solutions containing 1000 mg L^−1^ of Ag(I), Al(III), As(III), B(III), Ba(II), Be(II), Bi(III), Ca(II), Cd(II), Co(II), Cr(III), Cu(II), Dy(III), Er(III), Eu(III), Fe(III), Ga(III), Ge(IV), Hg(II), Ho(III), I(-I), In(III), Ir(III), K(I), Li(I), Mg(II), Mn(II), Na(I), Nb(V), Ni(II), Os(IV), Pb(II), Pd(II), Pr(III), Pt(IV), Rb(I), Rh(III), Sb(III), Sc(III), Se(IV), Sn(II), Sr(II), Tb(III), Tl(I), V(V), Y(III), or Zn(II) (Sigma-Aldrich, Darmstadt, Germany) were used to prepare the working standard solutions. A CPC–505 pH-meter (Elmetron, Poland) was used to measure the pH of the solutions.

## Results and discussion

### Optimization of the working parameters

#### Carrier gas flow rate

**Fig. 2:**
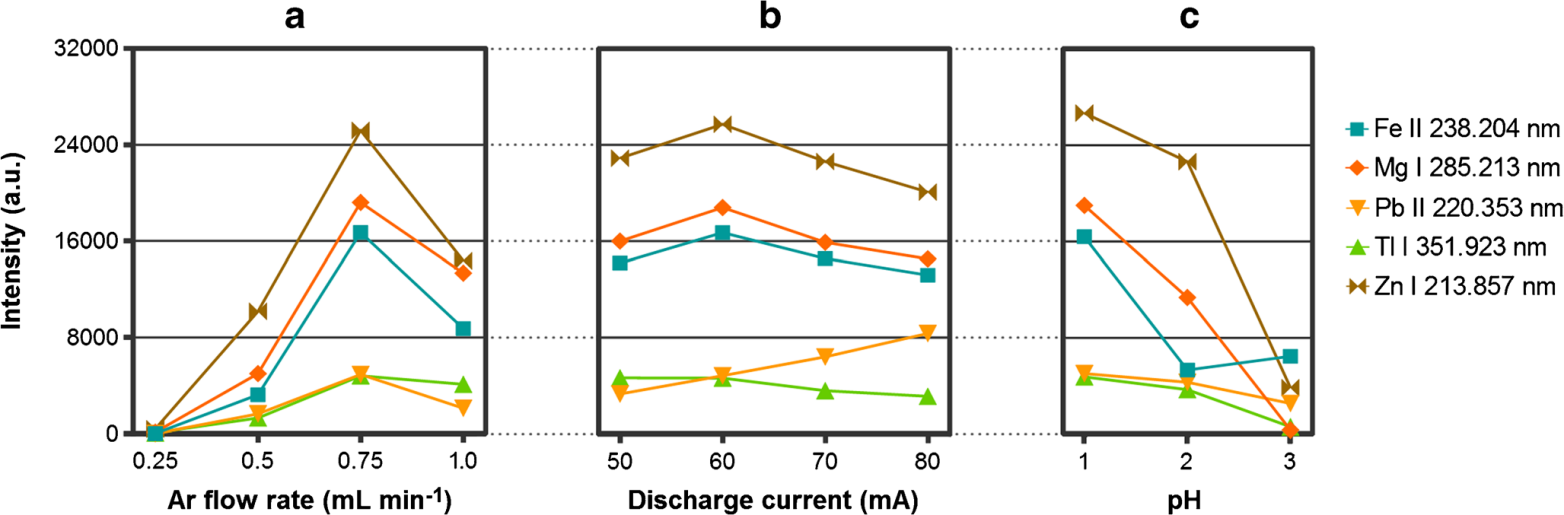
The effect of (A) the Ar flow rate, (B) the discharge current, and (C) the sample solution pH on the intensity of the Fe, Mg, Pb, Tl, and Zn emission lines obtained in HDC-APGD-ICP-OES

In the first step, the effect of the carrier gas (Ar) flow rate on the response of different elements was investigated. A multi-element solution of Fe, Mg, Pb, Tl, and Zn (each element at 1 mg L^−1^) acidified with HNO_3_ to pH = 1 was introduced to the HDC-APGD system operated at a discharge current of 60 mA. The produced aerosol flux was swept by Ar (flow rate in the range of 0.25–1.0 L min^−1^) to the ICP torch, and the response of analytes was acquired. As can be seen from Fig. 2a, for all of the examined elements, the highest analytical response was obtained at a flow rate of 0.75 L min^−1^. Below this value, the response of the studied elements was drastically suppressed. It was also found that at the lowest studied carrier gas flow rate (0.25 L min^−1^), the aerosol flux produced by HDC-APGD condensed inside the U-tube condenser and the cyclonic chamber to a greater extent. On the other hand, when the Ar flow rate exceeded 0.75 L min^−1^, the residence time of the analytes in ICP was shortened, which resulted in lower emission from the studied elements. The effect of the carrier gas flow rate in the sample introduction system equipped with the OneNeb nebulizer and the cyclonic spray chamber was also studied for comparison. Despite the differences in the mechanism of the aerosol flux formation in the HDC-APGD system (e.g., water evaporation, cathodic sputtering of the solution components) and the PN system, the effect of the carrier gas flow rate for both systems was quite similar. The highest response of the studied elements was obtained at a flow rate equal to 0.75 L min^−1^ (see ESM Fig. S1), which was the value recommended by the nebulizer manufacturer. Nevertheless, in the case of HDC-APGD, intensities of the analytical lines were, on average 2 times higher, which justified the use of this novel sample introduction system.

#### Discharge current

Stable HDC-APGD was operated at the discharge current in the range of 50–80 mA. The greater amperage applied, the higher sample flow rate was needed to compensate for the evaporation of the sample solution, being the cathode of the discharge system. The sample consumption at 50, 60, 70, and 80 mA were respectively 0.40, 0.56, 0.70, and 0.94 mL min^−1^. APGD could be sustained below 50 mA, but there was no self-ignition of the discharge, which made it difficult to use the developed closed HDC-APGD system combined with the ICP spectrometer. As the discharge current exceeded 80 mA, the discharge was quite unstable, and instead of the formation of the fine aerosol, large droplets were rejected from the HDC solution. To study the effect of the discharge current in more detail, a multi-element solution of Fe, Mg, Pb, Tl, and Zn (1 mg L^−1^) acidified with HNO_3_ to pH = 1 was introduced to HDC-APGD and the emission from analytes was recorded. As can be seen from Fig. 2b, in the case of Pb, the highest emission was noted at 80 mA, which likely resulted from the enhanced flux of the analytes atoms reaching ICP. For Fe, Mg, Tl, and Zn, the highest analytical signals were obtained at an apparently lower discharge current, i.e., 60 mA. Although at a lower amperage the flux of analytes atoms decreased, the amount of the water vapor introduced into the ICP torch was also reduced, and hence, higher sensitivity was achieved. Therefore, the discharge current of 60 mA was recognized as the most beneficial for a wider group of elements and considered as optimal for further work.

#### Sample acidity

The response of studied elements in HDC-APGD-ICP-OES was monitored as a function of the sample pH in the range of 1–3 (adjusted with concentrated HNO_3_). It was revealed that an increase in pH suppressed remarkably the analytical signals (Fig. 2c). The obtained results coincided with those reported for ELCAD [17–19] and LS-APGD [20], where pH of the liquid cathode was established to play a crucial role in releasing the analytes atoms from the sample solution through the cathodic sputtering. Accordingly, at a higher acid concentration, the sputtering efficiency increased, resulting in the boosted response of analytes [17–19]. In the discharge system presented herein, the introduced sample solution was totally consumed, and hence, changes in its pH could not affect the sputtering efficiency. Nevertheless, it could be presumed that a decrease of the pH resulted in a decrease of the cathodic fall of the HDC, leading to the improvement of the emission rate of secondary electrons [21]. These secondary electrons could likely take part in collisions and collision ionization processes in the gaseous phase of the discharge, being responsible for improved excitation conditions of the HDC-APGD. Since this gaseous phase of the discharge was introduced into a central channel of the ICP torch, higher analytical signals were observed in these conditions as a consequence of the higher sample acidity. Indeed, despite the differences between HDC-APGD and ELCAD, the effect of the sample acidity in both discharge systems was quite similar. It is worth to mention that in the case of HDC-APGD, apparent emission from analytes was also observed at pH > 3. However, to provide sufficient electrical conductivity, NH_4_NO_3_ had to be added to the sample solution, and the resulted analytical signals were lower than those acquired at pH = 1.

Summarizing, the Ar flow rate of 0.75 L min^−1^, the discharge current = 60 mA, the corresponding sample uptake rate of 0.56 mL min^−1^, and the sample pH = 1 were recognized to provide the most favorable conditions for the sample solution introduction to the ICP torch with the HDC-APGD system for most of the studied elements. These conditions were maintained during all further experiments. As compared to the PN system, the developed HDC-APGD system offered on average 2-fold higher intensities of the emission lines of studied analytes.

### Transport efficiency of analytes

In order to explain the enhancement of the response of analytes in ICP-OES resulting from HDC-APGD, the nebulization performance of this novel system due to the cathodic sputtering and/or the thermal evaporation phenomena was evaluated. It was obtained by a comparison of the nominal concentration of elements in the sample solution before its introduction to HDC-APGD, and the concentration of these elements determined in the waste solution collected from the U-tube condenser and the cyclonic chamber. The introduced sample solution was a multi-element standard solution of Ag, Al, B, Cd, Co, Cr, Cu, Fe, In, K, Li, Mg, Mn, Na, Ni, Tl, and Zn (at 1 mg L^−1^). Based on the mass loss of the waste solution versus the introduced sample solution, the percentage of water introduced to ICP was determined to be 17 ± 1%, and this value was included in further calculations of transport efficiencies of analytes. Concurrently, intensities of analytical lines of the studied elements measured with HDC-APGD-ICP-OES were acquired and compared to those obtained with PN-ICP-OES. The results of the transport efficiency of investigated elements and intensities of their emission lines obtained with the developed HDC-APGD system, given as ratios in reference to intensities of these lines obtained with the PN system, are listed in Table 1.

**Table 1:** The transport efficiencies of analytes in the HDC-APGD system (*n* = 3, ±SD), and ratios of intensities of analytical emission lines obtained using HDC-APGD-ICP-OES versus intensities of these lines obtained using PN-ICP-OES

Element	The efficiency of sputtering and transport (%)	Intensity ratio
Ag	89.6 ± 0.5	0.63
Al	71.8 ± 0.9	2.83
B	20.5 ± 5.9	0.056
Cd	86.7 ± 3.4	0.89
Co	95.6 ± 3.9	2.92
Cr	84.4 ± 3.8	3.13
Cu	81.1 ± 6.1	0.65
Fe	42.4 ± 0.5	2.85
In	84.5 ± 4.6	2.16
K	49.5 ± 12.1	3.06
Li	84.0 ± 4.1	2.23
Mg	73.4 ± 1.1	2.83
Mn	83.5 ± 4.0	3.12
Na	53.4 ± 8.2	2.64
Ni	83.9 ± 9.9	2.93
Tl	81.2 ± 3.6	1.80
Zn	32.5 ± 6.6	2.74

For most of the elements, the transport efficiency of analytes in the investigated sample introduction system was in the range of 70–90%. Assuming that the PN efficiency is ~ 5–10%, the aerosol flux produced by HDC-APGD and the respective amount of analytes was about 10 times higher. Given this fact, it could be expected that analytical signals acquired with HDC-APGD-ICP-OES should also be enhanced by one order of magnitude. Surprisingly, in the case of most studied elements, intensities of their emission lines were boosted only by a factor of 2–3 (as compared to those obtained with PN-ICP-OES). Moreover, for Ag, B, Cd, and Cu, the emission from these elements in HDC-APGD-ICP-OES was even lower than those observed in PN-ICP-OES. The most likely reason could still be a too high water flux introduced from HDC-APGD into ICP, i.e., 0.095 g min^−1^, that was approximately 2.5 times higher as compared to the PN-ICP system, i.e., 0.038 g min^−1^ (assuming the nebulization efficiency of 5%). An increased amount of water getting into the central channel of ICP could significantly deteriorate the excitation conditions thereof [22]. Hence, it would be reasonable to miniaturize the subsequent HDC-APGD arrangements to reduce the sample uptake rate and the resulting water flux. The strength of the developed system was that the relative amounts of analytes atoms introduced to the ICP, i.e., 70–90%, were about 5 times higher than the relative amount of water (17%). In other words, the produced aerosol flux was significantly enriched in analytes, resulting in the improvement of their signals acquired with ICP-OES.

### Comparison of HDC-APGD and PN

To prove the applicability of the HDC-APGD system in a multi-element analysis by ICP-OES, the group of studied elements was expanded, and the intensities of emission lines of 47 elements were determined and compared with those obtained for conventional PN-ICP-OES (see Table 2, results given as ratios). For this purpose, a series of multi-element standard solutions was prepared; all standard solutions were acidified with HNO_3_ to pH = 1.0 and the concentration of all individual elements was 1 mg L^−1^. Resulting solutions were introduced one by one into ICP-OES using the HDC-APGD system or the PN system, and the intensities of the most prominent emission lines of elements were measured.

**Table 2:** Comparison of the emission intensity in ICP-OES operated with a hanging drop cathode atmospheric pressure glow discharge (HDC-APGD) system and a pneumatic nebulizer (PN) (given as a ratio), with/without the addition of different LMWOCs

Element	Wavelength (nm)	Intensity ratio (HDC-APGD-ICP-OES/PN-ICP-OES)					
		LMWOCs addition (2%, m/m)					
		None	CH_3_OH	C_2_H_5_OH	HCOOH	CH_3_COOH	HCHO
Ag I	328.1	0.63	0.86	0.85	0.89	1.1	0.74
Al I	396.2	2.8	1.7	1.6	1.6	1.5	1.6
As I	193.7	2.6	1.6	1.3	1.6	1.7	1.6
B I	249.7	0.056	0.27	0.23	0.14	0.22	0.28
Ba II	455.4	2.9	1.8	1.6	1.8	1.6	1.7
Be I	234.9	1.6	1.4	1.8	1.8	1.5	1.5
Bi I	223.1	0.76	0.79	0.77	1.6	0.84	1.4
Ca I	422.7	2.6	1.6	1.7	1.9	1.7	1.7
Cd II	226.5	0.89	1.2	0.92	1.1	1.0	1.4
Co II	238.9	2.9	1.8	1.7	1.8	1.5	1.7
Cr II	267.7	3.1	2.0	1.8	1.9	1.7	1.8
Cu I	327.4	0.65	0.7	0.71	0.63	0.68	0.66
Dy II	353.2	1.6	1.3	1.4	1.6	1.4	1.4
Er II	349.9	1.2	0.92	1.1	1.2	1.1	1.0
Eu II	420.5	1.6	1.2	1.4	1.6	1.4	1.3
Fe II	238.2	2.9	1.8	1.7	2.0	1.7	1.8
Ga I	294.4	2.8	1.6	1.6	1.6	1.5	1.6
Ge I	265.1	1.8	1.6	1.7	1.8	1.6	1.6
Hg II	194.1	1.9	6.7	6.5	8.6	6.2	4.3
Ho II	345.6	1.6	1.3	1.4	1.6	1.4	1.4
I I	178.2	6.2	4.1	4.7	5.0	4.8	1.4
In I	325.6	2.2	1.2	1.1	1.1	1.0	1.4
Ir II	212.7	1.3	1.2	1.2	1.3	1.2	1.3
K I	766.5	3.1	1.9	1.9	1.7	1.6	1.8
Li I	670.8	2.2	1.6	1.9	1.7	1.6	1.8
Mg I	285.2	2.8	1.5	1.5	1.5	1.4	1.5
Mn II	257.6	3.1	2.0	1.7	1.8	1.6	1.8
Na I	589.6	2.6	1.6	1.7	1.6	1.5	1.8
Nb II	269.7	2.3	2.0	2.3	2.5	2.2	2.0
Ni II	231.6	2.9	2.0	1.8	1.9	1.7	1.8
Os II	225.6	0.72	0.86	0.63	1.4	1.1	0.56
Pb II	220.4	0.80	0.92	0.89	1.3	0.87	0.93
Pd I	340.5	0.43	0.43	0.35	0.38	0.29	0.52
Pr II	410.1	1.6	1.3	1.5	1.6	1.4	1.4
Pt I	214.4	0.50	0.57	0.49	0.53	0.34	0.52
Rb I	780.0	1.8	1.6	2.1	2.0	1.6	1.7
Rh I	343.5	0.52	0.50	0.46	0.49	0.40	0.67
Sb I	206.8	2.5	1.6	1.5	1.4	1.7	1.8
Sc II	361.4	1.7	1.4	1.6	1.8	1.5	1.5
Se I	196.0	1.2	1.7	2.3	1.4	2.1	2.2
Sn I	284.0	2.7	1.7	1.5	1.5	1.3	1.7
Sr II	407.8	3.1	1.8	1.6	1.8	1.6	1.7
Tb II	350.9	1.6	1.3	1.5	1.6	1.4	1.4
Tl I	351.9	1.8	1.3	1.3	1.4	1.3	1.4
V II	292.4	0.46	0.4	0.47	0.5	0.44	0.43
Y II	371.0	6.1	5.0	5.8	6.3	5.5	5.3
Zn I	213.9	2.7	1.8	1.7	1.7	1.7	1.7

As shown in Table 2, depending on the element, the obtained intensity ratios varied from 0.056 (in the case of B) to about 6 (in the case of I and Y). For some selected elements (Ag, Cd, Fe, Hg, I, Mg, Os, Pb, Tl, Zn), the calibration curves for 0.01, 0.1, and 1 mg L^−1^ standards solutions were acquired by ICP-OES using both studied sample introduction systems, i.e., HDC-APGD and PN. The slopes and intercepts of these calibration curves as well as their determination coefficients (*R*^2^) achieved for HDC-APGD-ICP-OES are given in ESM Table S2. In addition, the ratios of the slops of the calibrations curves obtained using the HDC-APGD system to those obtained with the PN system are given. They well corresponded to the intensity ratios found for both studied systems. The low response of B strictly corresponded to the low efficiency of its transport to ICP (see Table 1). This observation was in line with the results obtained by Cserfalvi et al. [12] who also studied the sample introduction system based on ELCAD. In general, as compared to ELCAD-ICP-OES, the response of elements in HDC-APGD-ICP-OES was an order of magnitude higher, which likely resulted from the improved sputtering and/or evaporation of the sample solution. It should be noted that the analyzed samples were totally consumed by the discharge [17]. Cserfalvi et al. [12] found that in ELCAD-ICP-OES, Hg showed a “super-sputtering” effect, resulting in its 17 times higher signal as compared to this acquired with PN. Herein, the sensitivity of Hg was improved only 2 times, and it was comparable with this observed for other elements. The likely explanation for this could be an increased amount of the water vapor that was introduced into ICP (in comparison to ELCAD). Besides a noticeable exception for Hg, it seems that the behaviour of other elements in HDC-APGD and ELCAD was quite similar. The highest improvement of the analytical response in HDC-APGD-ICP-OES was observed for I and Y. Because of no literature data, the results obtained for Y are challenging to explain. As for I, its high response is consistent with the results obtained for ICP-OES coupled with a similar microplasma system, i.e., SCGD, reported by Zhu et al. [15]

In general, using the HDC-APGD system, intensities of emission lines of studied elements were on average 2 times higher (as compared to PN-ICP-OES). For 36 out of 47 studied elements, the use of HDC-APGD provided enhanced analytical signals which proved the usability of this novel system in multi-element analysis by ICP-OES.

To further improve the analytical performance of HDC-APGD-ICP-OES, the effect of the addition of low molecular weight organic compounds (LMWOCs) into the sample solution was also investigated. According to the literature data, it seems that LMWOCs promote the formation of volatile derivatives of analytes in APGD-type systems, which results in the significant enhancement of their signals [23–28]. Herein, the following LMWOCs were examined: alcohols (CH_3_OH, CH_2_H_5_OH), acids (HCOOH, CH_3_COOH), and aldehyde (HCHO). As previously, a series of multi-element standard solutions was prepared. Each standard solution was acidified with HNO_3_ to pH = 1.0 and contained several elements that did not interfere to each other. Moreover, individual LMWOCs were added separately at a concentration of 2% (m/m), which was recognized to be the highest value that did not destabilize ICP.

As can be seen from Table 2, in the presence of LMWOCs, emission from 8 elements, i.e., Ag, B, Bi, Cd, Hg, Os, Pb, and Se, was improved at least by 50% (as compared to results without organic additives). Significantly, almost all of these elements (except for B) are known to readily form different volatile species, e.g., cold vapors, hydrides, and oxides, which supports the thesis that LMWOCs promote the formation of volatile derivatives. The highest signal improvement, i.e., 8.6 times, was observed for Hg in the presence of HCOOH, which was in agreement with the results obtained for the sample introduction system based on SCGD described by Zhu et al. [13]. In the last step, the background intensity and its standard deviation (SD) in the vicinity of the I 178.2 nm, Hg 194.1 nm, Zn 213.9 nm, Fe 238.2 nm, Mg 285.2 nm, and Tl 351.9 nm emission lines were estimated (see Table 3). It was revealed that for HDC-APGD-ICP-OES the background intensity and its SD were respectively, on average, 2.7 and 2.2 times higher, as compared to those obtained for conventional PN-ICP-OES. As such, 2 times signal improvements were offseted by a 2 times increase in SD of the background (noise). However, for some elements, e.g., Hg and I, the enhancement of the signal intensity was higher than the noise increase, resulting in the improvement of LODs of these elements. The most likely reason for the observed increase in the background intensity and its SD was still a high water load into the ICP. In our opinion, a further effort should be directed to miniaturize the discharge system, which would improve the analytical performance of HDC-AGD-ICP-OES, and additionally, minimize the sample consumption. The use of a more efficient condenser could also help to introduce smaller amounts of the water vapor accompanying the discharge.

**Table 3:** A comparison of the background intensity and its standard deviation in the vicinity of different emission lines for ICP-OES combined with a hanging drop cathode-atmospheric pressure glow discharge (HDC-APGD) system and a nebulizer/cyclonic spray chamber system for pneumatic nebulization (PN)

Line (nm)	Average background intensity (counts)			Background standard deviation (counts)		
	HDC-APGD	PN	HDC-APGD/PN	HDC-APGD	PN	HDC-APGD/PN
I (178.2)	208	98	2.1	14.7	6.54	2.2
Hg (194.1)	1225	291	4.2	58.6	17.4	3.4
Zn (213.9)	2356	911	2.6	34.4	25.4	1.4
Fe (238.2)	896	207	4.3	56.2	27.5	2.0
Mg (285.2)	6849	3886	1.8	83.9	38.6	2.2
Tl (351.9)	6027	4451	1.4	161.5	80.0	2.0

## Summary and conclusions

In comparison to conventional PN-ICP-OES, HDC-APGD-ICP-OES offered on average 2 times higher analytical signals of elements. The developed system was beneficial in the case of 41 investigated elements (Ag, Al, As, Ba, Be, Bi, Ca, Cd, Co, Cr, Dy, Er, Eu, Fe, Ga, Ge, Hg, Ho, I, In, Ir, K, Li, Mg, Mn, Na, Nb, Ni, Os, Pb, Pr, Rb, Sb, Sc, Se, Sn, Sr, Tb, Tl, Y, and Zn), and the most remarkable improvements were achieved for Hg, I, and Y. The boosted response of elements could be assigned to their improved transport efficiency. In the case of some elements, > 80% of their initial amount (supplied to HDC-APGD) was converted to the respective aerosol flux, and introduced into the ICP torch. The amount of the water vapor concomitantly introduced into ICP was recognized to be a critical parameter limiting the performance of the developed system. A too high flux of the water vapor was partially reduced by the use of a simple U-tube condenser. However, as to further development of HDC-APGD, succeeding constructions should be miniaturized, which would reduce the sample solution consumption. Alternatively, the use of a more efficient cooling block should be considered. This outline paper laid only the groundwork for the development of a new sample introduction technique, and a more in-depth study is needed. Nonetheless, the presented results gave a credence to the fact that HDC-APGD is a promising and high-performance sample introduction system.

## Electronic supplementary material

ESM 1 (PDF 175 kb).

## Data Availability

All data generated or analyzed during this study are included in this published article and its supplementary information file.
