# Supplementary material for: Hanging drop cathode-atmospheric pressure glow discharge as a new method of sample introduction for inductively coupled plasma-optical emission spectrometry
Source: Anal Bioanal Chem. 2020 May 11;412(18):4211–9. doi: 10.1007/s00216-020-02685-7 (PMC7320056; doi:10.1007/s00216-020-02685-7)
Supplement: Supplementary file 1 — (PDF 175 kb). [file 216_2020_2685_MOESM1_ESM.pdf]

Analytical and Bioanalytical Chemistry

Electronic Supplementary Material

Hanging drop cathode - atmospheric pressure glow discharge as a new method of sample introduction for inductively coupled plasma - optical emission spectrometry

Krzysztof Swiderski, Maja Welna, Krzysztof Greda, Pawel Pohl, Piotr Jamroz

Overview: Supporting information containing the operating parameters of the ICP-OES spectrometer working with pneumatic nebulization (PN) (Table SI-1), the slopes and intercepts of calibration curves achieved for selected elements with the aid of the HDC-APGD-ICP-OES system, and the influence of the carrier gas (Ar) flow rate on intensities of Fe, Mg, Pb, Tl , and Zn emission lines in ICP-OES with pneumatic nebulization (PN).

Table S1 The operating parameters of the ICP-OES spectrometer with pneumatic nebulization (PN)

Parameter	Setting
Plasma torch	Horizontally oriented, quartz, one-piece with a 2.4 mm injector
Sample introduction system	Glass, one-pass cyclonic chamber with an OneNeb nebulizer
Optical/detection system	High-resolution Echelle-type, 400 mm focal length thermostated polychromator with a cooled cone interface (CCI), and a custom-designed, image mapping (IMAP) and adaptive integration (AIT) technologies featuring CCD detector
Supplied RF power	1.2 kW
Plasma Ar flow rate	15.00 L min^{-1}
Auxiliary Ar flow rate	1.5 L min^{-1}
Nebulizing Ar flow rate	0.75 L min^{-1}
Sample uptake rate	0.75 mL min^{-1}
Stabilization delay	15 s
Solution uptake delay	30 s
Replicate read time	1 s
Replicates	3
Rinse time	10 s

Table S2 The slopes and intercepts of calibration curves achieved for selected elements with the aid of ICP-OES combined with a hanging drop cathode atmospheric pressure glow discharge (HDC-APGD) system and a pneumatic nebulizer/spray chamber (PN) system (given as a ration)

Line, nm	Slope, a. u./mg L^{-1}	Intercept, a. u.	R^2	$Slope_{HDC-APGD}/Slope_{PN}$
Ag I 328.1	8.06×10^3	0.05×10^3	0.99970	0.69
Cd II 226.5	2.67×10^5	0.02×10^5	0.99880	0.89
Fe II 238.2	1.70×10^5	0.001×10^5	0.99999	2.72
Hg II 194.1	2.32×10^4	0.04×10^4	0.99999	2.21
I I 178.2	2.40×10^3	0.07×10^3	0.99820	6.15
Mg I 285.2	6.98×10^5	0.05×10^5	0.99980	2.54
Os II 225.6	2.75×10^3	0.04×10^3	0.99960	0.76
Pb II 220.4	6.57×10^4	0.08×10^4	0.99990	0.82
Tl I 351.9	4.46×10^4	0.04×10^4	0.99990	2.01
Zn I 213.9	2.58×10^5	0.002×10^5	0.99990	2.88

R^2 Determination coefficient.

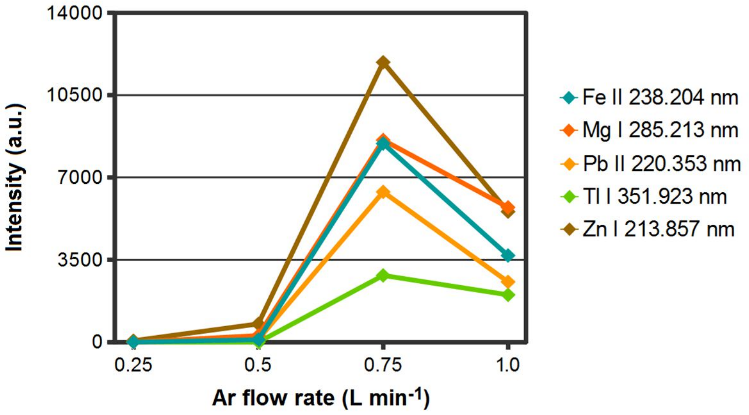

Fig. S1 The effect of the carrier Ar flow rate on the intensity of the Fe, Mg, Pb, Tl, and Zn emission lines obtained for ICP-OES with pneumatic nebulization (PN)
